# Evaluation of Non-linear Models to Predict Potential Milk Yield of Beef Cows According to Parity Order Under Grazing

**DOI:** 10.3389/fvets.2021.721792

**Published:** 2021-11-23

**Authors:** Matheus Fellipe de Lana Ferreira, Luciana Navajas Rennó, Isabela Iria Rodrigues, Sebastião de Campos Valadares Filho, Luiz Fernando Costa e Silva, Fabyano Fonseca e Silva, Edenio Detmann, Mário Fonseca Paulino

**Affiliations:** Department of Animal Science, Universidade Federal de Viçosa, Viçosa, Brazil

**Keywords:** milk production, Bos indicus, lactation, beef cattle, milk composition

## Abstract

This study aimed to evaluate the effect of parity order on milk yield (MY) and composition over time of grazing beef cows and to evaluate non-linear models to describe the lactation curve. Thirty-six pregnant Nellore cows (12 nulliparous, 2 years; 12 primiparous, 3 years; and 12 multiparous, 4–6 years) were included in the study. With calving day assigned as day 0, milking was performed using a milking machine to estimate MY on days 7, 14, 21, 42, 63, 91, 119, 154, and 203. Dummy variable analyses were applied to estimate its effects on MY, composition (kg and percentage), afternoon/morning, and afternoon/total proportions. Since multiparous cows had higher MY than nulliparous and primiparous cows, two different groups were used for lactation curve analysis: Mult (multiparous) and Null/Prim (nulliparous and primiparous). The MY estimated by the last edition of BR-Corte (Nutrient Requirements of Zebu and Crossbred Cattle) equation was compared with the observed values from this study. Five nonlinear models proposed by Wood (WD), Jenkins & Ferrell (JF), Wilmink (WK), Henriques (HR) and Cobby & Le Du (CL) were evaluated. Models were validated using an independent dataset of multiparous and primiparous cows. The estimates for parameters a, b, and c of the CL equation were compared between groups, and the BR-Corte equation used the model identity methodology. Nulliparous and primiparous cows displayed similar MY (*P* > 0.05); however, multiparous cows had an average MY that is 0.70 kg/day greater than that of nulliparous and primiparous cows (*P* < 0.05). Milk protein and total solids were higher for multiparous cows (*P* < 0.05). Effect of days in milking was found for milk fat, protein, and total solids (*P* < 0.05). The yield of all milk components was higher for multiparous cows than for nulliparous and primiparous cows. The afternoon/morning and afternoon/total proportions of milk production were not affected by parities and days in milking (*P* > 0.05), with an average of 0.76 and 0.42, respectively. The BR-Corte equation did not correctly estimate the MY (*P* < 0.05). The equations of WD, WK, and CL had the best estimate of MY for both Mult and Null/Prim datasets. The equations had a very similar Akaike's information criterion with correction and mean square error of prediction.

## Introduction

The milking ability of beef cows is one of the main factors influencing the weaning weights of calves (1); thus, many methods have been used to attempt to estimate beef cows' milk production and its influence on calf preweaning growth (2–4). The evaluation of milk production is also necessary to estimate the nutrient requirements of cows and calves since the nutritional balance of a lactating cow is important in the calculation of energy and protein requirements (5, 6). Thus, if it is overestimated or underestimated, systematic errors can impair the estimation of nutrient requirements.

Milk yield can be estimated by different methods including determining differences in calf weights before and after suckling (2, 7, 8) and hand milking (3, 4) or machine milking procedures (9–11). Those methods are possible with small numbers of animals but are not adaptable to larger herds. Such procedures, particularly those with a controlled suckling period before calf separation as described by Boggs et al. (1), require repeated, intensive animal handling in which timing is critical. Furthermore, in general, data collection and handling procedures of grazing animals, specifically pregnant Nellore cows, are laborious since animals have a poor temperament and require extreme care. This challenge would explain the low number of studies and the low number of data points to fit an equation of milk production for grazing beef cows.

In the last BR-Corte edition [third edition of the Brazilian tables of *Nutrient Requirements of Zebu and Crossbred Cattle*; (12)], the estimated milk yield for Nellore cows during 7 months of lactation was based on the model proposed by Cobby and Le Du (CL) (13). The equation was generated using data from feedlot cows, but the authors employed independent data from experiments with grazing Nellore cows to validate the equation. Although the equation provided a better estimate of milk yield than did previous editions (14), none of these experiments explicitly evaluated the milk yield of different parity orders under grazing conditions, and there are still limited data in this regard.

Previous studies have shown that age and parity order can influence the metabolism and milk production of dairy (15, 16) and beef cows (6, 17), where primiparous cows display lower milk production and a more unbalanced nutritional status compared to multiparous cows (15, 17, 18). Thus, different equations are needed to predict the milk yield of Nellore cows according to parity order, since they may differ in milk potential.

Therefore, this study aimed to evaluate the effect of parity order on milk yield and composition over time of grazing beef cows and to evaluate non-linear models to describe the lactation curve.

## Materials and Methods

The Animal Care and Use Committee at the Universidade Federal de Viçosa (UFV), Brazil (protocol CEUAP-UFV 120/2018), approved all animal care and handling procedures. Animals used in this study were provided by the UFV/Beef Cattle Research and Extension Unit, Viçosa-MG, Brazil, where the study was conducted from July 2018 to May 2019.

### Experimental Design and Animals

Thirty-six pregnant Nellore cows (12 nulliparous, 12 primiparous, and 12 multiparous) were included in the study, with the following average age, body weight (BW), and body condition score (BCS; 1–9): 2 years, 442 (±62) kg, 6.20 (±0.5); 3 years, 457 (±58) kg, 5.68 (±0.5); and 4–6 years, 505 (±60) kg, 5.92 (±0.5), respectively. This study lasted from 60 days prepartum to 203 days of the lactation period (2 weeks before weaning).

The nomenclature for each category related to parity was set at the beginning of the experiment (late gestation period) and used throughout the manuscript. Even though after calving the parity order changed (e.g., nulliparous cows became primiparous), the nomenclature remained constant along the manuscript.

Parity classes were systematically randomized into six paddocks, assuming two cows from each parity class in the paddocks (thus characterizing sub-blocks). The animals were assigned to paddocks 15 days before the beginning of the experiment to acclimate them to the environment and the herd. The average area of the paddocks was 7 ha, covered with *Urochloa decumbens* grass, and cows had free access to water and feeders.

All cows were group-fed with an energy-protein supplement (1.0 kg/day) with 35% crude protein (CP) for 60 days prepartum (41.2% corn meal, 56.3% soybean meal, and 2.5% urea: ammonium sulfate). The supplement was calculated to supply approximately 40% of the cows' protein requirements, as recommended by the BR-Corte (12). We provided a linear trough space of 0.70 m per cow to ensure homogeneous supplement consumption among groups. The supplement was supplied at 12:00 pm to minimize any interference from animal grazing behavior.

After calving, cows remained in the same paddocks with their respective calves. A commercial mineral mix (CaHPO_4_ = 50.00%; NaCl = 47.775%; ZnSO_4_ = 1.4%; Cu_2_SO_4_ = 0.70 %; CoSO_4_ = 0.05%; KIO_3_ = 0.05%; and MnSO_4_ = 0.025%) was also offered to cow–calf pairs for *ad libitum* consumption throughout the experiment, supplied separately in additional feeders.

After 90 days of age, calves were offered 5 g/kg BW of an energy-protein supplement formulated to contain 20% CP in a creep-feeding system until the end of the study (203 days in milk).

### Milk Collection and Analyses

Calving day was assigned as day 0, and milking was performed using a milking machine to estimate milk yield on days 7, 14, 21, 42, 63, 91, 119, 154, and 203.

Milking procedures were as described by Boggs et al. (1), providing a controlled suckling period before the calf separation. To empty udders, calves were separated from their dams at 3:00 pm and then reunited at 5:45 pm and allowed to suckle until 6:00 pm, when they were once again separated. The first milking was performed at 6:00 am on the next day after an injection of 10 IU (international unit) of oxytocin (10 IU/ml; Ocitovet®, Brazil) in the cow's mammary vein, and the produced milk was weighed. The exact time when the milking of each cow ended was recorded. After morning milking, cows were kept separated from their calves until the end of the afternoon milking, which was performed at 6:00 pm. Then, the total daily production was calculated by the sum of both milking times to obtain a 24-h milk production.

From each cow, a 30-ml sample of milk was collected at morning and afternoon milking to evaluate milk composition. Samples were stored at 4°C in a refrigerator, each receiving one bronopol tablet per sample as a preservative for further analyses. Milk samples were analyzed fresh for percentage of protein, fat, lactose, and total solids content using infrared spectroscopy (Foss MilkoScan FT120, São Paulo, Brazil). A weighted average was calculated for each component based on morning and afternoon milk yields.

### Forage Sampling and Analyses

Every 30 days, grass samples were collected by hand-plucking to evaluate the forage selected by animals and collected by cutting at the ground level from five delimited areas (0.5 × 0.5 m), selected randomly in each paddock to quantify dry matter (DM) per hectare. In these circumstances, all the samples were weighed, oven-dried (55°C), and then ground to pass through 1- and 2-mm screens in a Wiley mill (model 3, Arthur H. Thomas, Philadelphia, USA). All data from each month were combined and expressed as an average per season as follows: dry season = August (beginning of the experiment), dry–rainy transition = September to November; rainy season = December to February; and rainy–dry transition = March to June (end of the experiment).

The forage and supplement samples were analyzed following the procedures described by the Brazilian National Institute of Science and Technology in Animal Science [INCT-CA; (19)] for DM (method G-003/1), ash (method M-001/1), CP (method N-001/1), and neutral detergent fiber corrected for ash and protein (apNDF; method F-002/1). Indigestible neutral detergent fiber [iNDF; (20)] was processed at 2 mm and quantified by *in situ* incubation procedures with non-woven textile bags (100 g/m^2^) for 288 h.

### Statistical Analyses

Variables measured during lactation were analyzed using the following model:


(1) $$\begin{array}{l} Y_{ijkl}=\mu +{C_{j}+P}_{k\left(l\right)}+e_{ijkl} \end{array}$$

where *Y*_*ijkl*_ = observation measured on animal *i*, belonging to parity class *j*, within paddock *k* and sub-block *l*; μ = overall mean; *C*_*j*_ = parity effect *j* (fixed); *P*_*k*__(l)_ = paddock effect *k* with the nested sub-block *l* (random); and *e*_*ijkl*_ = residual random effect, assumed to be independent and identically distributed (0, σ^2^_*e*_). The covariate day in milk was added to this model when its effect was significant.

Given the ordinal nature of parity class (0, 1, and 2), dummy variable analyses (21) were applied to estimate its effects on milk yield, composition, and afternoon/morning and afternoon/total proportions. For this, the mixed model presented in [1] was fitted by using PROC MIXED of SAS® software, assuming the dummy variable as covariates. The parity effect estimates were added to the overall mean to report the results on the same scale as the observed data.

Based on model [1], since multiparous cows had higher milk yield than nulliparous and primiparous cows, two different groups were used for lactation curve analysis: Mult (multiparous cows) and Null/Prim (both nulliparous and primiparous categories together).

The milk yield estimated by the BR-Corte (12) equation was compared with the observed values from this study using the Model Evaluation System (MES version 3.2.2) using the following regression model:


$$\begin{array}{l} y=\;\beta_{0}+\;\beta_{1}\times X \end{array}$$

where *x* = predicted values; *y* = observed values; and β_0_ and β_1_ = intercept and slope, respectively.

The regression was evaluated according to the following statistical hypothesis:


$$\begin{array}{l} H_{0}:\beta_{0}=0and\beta_{1}=1\;and\;H_{\text{a}}:notH_{0} \end{array}$$

Estimates were evaluated using the estimated value of the mean square error of the prediction and its components (22):


$$\begin{array}{l} MSEP=SB+MaF+MoF=\;1/n\;\sum_{i=1}{\left(x_{i}-y_{i}\;\right)}^{2},\\ \;SB={\left(x-y\right)}^{2},\\ \;MaF={\left(S_{x}+S_{y}\right)}^{2},\\ MoF=2S_{x}S_{y}\left(1-r\right), \end{array}$$

where *x* is the predicted values; *y* is the observed values; *MSEP* is the mean squared error of prediction; *SB* is the squared bias; *MaF* is the magnitude of random fluctuation; *MoF* is the model random fluctuation; *S*_*x*_ and *S*_*y*_ are the standard deviations of the predicted and observed values, respectively; and *r* is the Pearson linear correlation between the predicted and observed values.

For all calculations of variance and covariance, the total number of observations was used as a divisor because it was an estimate of the prediction error (22). The prediction of efficiency was determined by estimating the correlation and concordance coefficient (CCC) or reproducibility index described by Tedeschi (23).

The CCC indicates models with good accuracy and precision (when close to 1.0) or models with a problem of reproducibility (when close to 0.0). The smallest mean square error of prediction indicates the best model in the evaluation. In this study, it can indicate that the model error is associated with the squared bias (*SB*) or errors related to the high dispersion of data around the mean (*MaF*) or systematic errors concerning the direction of the curve predicted (*MoF*).

Five non-linear different equation forms were fitted to the datasets Mult and Null/Prim:

[CL] Cobby and Le Du (13) $Y_{t}=a+bt-a\exp^{\left(-ct\right)}+\;e_{t}$
[WD] Wood (24) $Y_{t}=a^{t}b\exp^{\left(-ct\right)}+\;e_{t}$
[WK] Wilmink (25) $Y_{t}=a-b\exp^{\left(-ct\right)}-dt+\;e_{t}$
[JF] Jenkins and Ferrell (26) $Y_{t}=at\exp^{\left(-ct\right)}+e_{t}$
[HR] Henriques et al. (27) $Y_{t}=a+bt\exp^{\left(-ct\right)}+\;e_{t}$

where *Y*_*t*_ = milk yield (kg/day) from lactating beef cows; *t* = time of lactation (weeks), exp = exponential of natural logarithm; *a* = theoretical initial yield (production scale) (WD, CL, WK, JF, and HR); *b* = decrease rate of production after peak of lactation (CL) and increase rate of production up to peak of lactation (WD, HR, and WK); *c* = increase rate of production up to peak of lactation (CL) and decrease rate of production after peak of lactation (WD, JF, and HR); *d* = decrease rate of production after peak of lactation (WK); and *e*_*t*_ = residual term, assumed as NIID (0, σ^2^_*e*_).

WD (24) is the most widely applied equation for dairy cattle lactation curve. JF (26) proposed a similar equation to WD but without the parameter *b*. The HR (27) equation is based on the JF model with the addition of a parameter for adjustment for the beginning of lactation. WK (25) is a modification of the lactation curve function in CL.

The parameters of the functions were estimated through the procedure NLMIXED of SAS (version 9.3, SAS Inst. Inc., Cary, NC) and were adjusted by the Gauss–Newton method.

An independent database (Rodrigues, 2021, unpublished data) of Nellore cows from the UFV/Beef Cattle Research and Extension Unit was used for training the models, which were composed of 130 and 112 observations of multiparous (*n* = 16) and primiparous (*n* = 14) cows, respectively, with eight lactation points each (1, 2, 4, 6, 9, 13, 20, and 29 weeks). The milking procedures were performed using the exact same approach as the present study: milking twice (morning and afternoon) with a controlled suckling period before the calf separation. The predicted values of the alternative equations generated were compared to the external data values of milk yield using MES (version 3.2.2). Akaike's information criterion with correction (AICC), MSEP, and the distribution of the error (SB, MaF, and MoF) were used to choose the best models.

Milk production upon lactation peak and time until peak were calculated for a 30-week lactation length by the derivative of the equations equal to zero.

The estimates for the parameters *a, b*, and *c* from the CL (13) model were compared between groups and the BR-Corte equation (12) (current equation to estimate milk yield of Nellore cows) using model identity methodology based on the overlapping of asymptotic confidence intervals (21).

All statistical evaluations were performed considering 0.05 as the critical level of probability for the occurrence of type I error.

## Results

**Figure 1:**
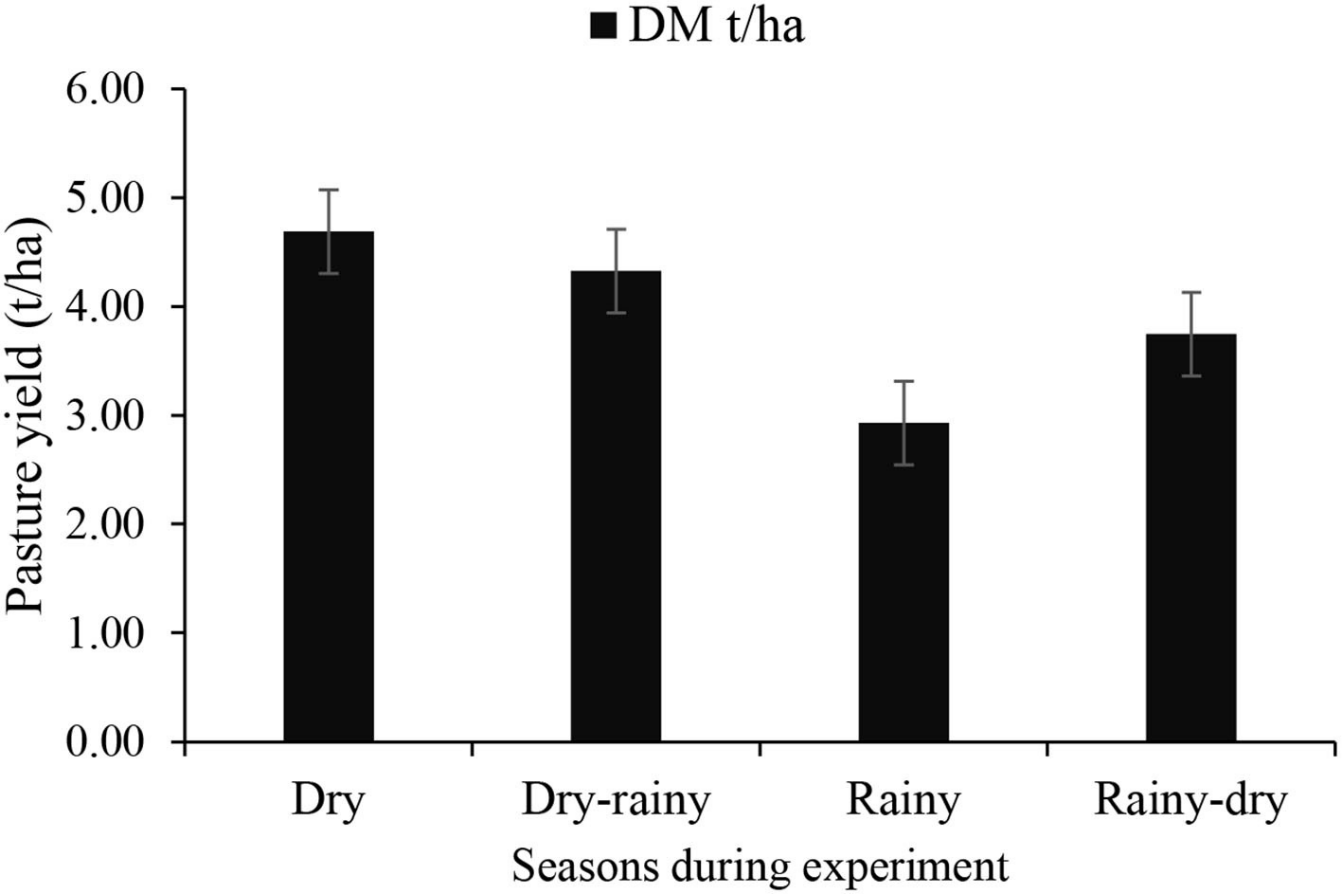
Pasture yield during experiment period per season. Dry season, August (beginning of the experiment); dry, rainy transition, September to November, rainy season, December to February; rainy–dry transition, March to June (end of the experiment). DM, dry matter; t/ha, ton per hectare.

The average DM yield was expressed per period (season) during the experiment as follows: dry season = 4.69 t/ha; dry–rainy transition = 4.33 t/ha; rainy season = 2.93 t/ha; and rainy–dry transition = 3.74 t/ha (Figure 1). Forage chemical composition by season is presented in Table 1.

**Table 1:** Forage chemical composition.

**Items**	***Urochloa decumbens***			
	**Dry**	**Dry–rainy**	**Rainy**	**Rainy–dry**
DM^1^	384.8	270.5	266.9	258.1
OM^2^	875.8	906.4	928.9	919.2
CP^2^	63.5	81.5	90.4	78.4
apNDF^2^	704.8	674.8	658.0	681.4
iNDF^2^	291.1	207.3	205.4	248.2
NDIN^3^	25.2	21.5	27.8	26.5

*DM, Dry matter; OM, organic matter; CP, crude protein; apNDF, neutral detergent fiber corrected for ash and protein; iNDF, indigestible neutral detergent fiber; NDIN, insoluble neutral detergent nitrogen*.

1 *g/kg of natural matter*,

2 *g/kg DM*,

3 *g/kg total nitrogen*.

Milk yield was different between parities (*P* < 0.05). Nulliparous and primiparous cows displayed similar milk yield (*P* > 0.05). However, multiparous cows had an average milk yield that is 0.70 kg/day greater than that of nulliparous and primiparous cows. The effect of days in milking was significant (*P* < 0.05) and was estimated as −0.008 kg/day, which is a decrease in production per day (Table 2).

**Table 2:** Regression coefficients for the covariate days in milk (with respective *P*-values), estimates of parity order effects and general standard error (SE).

**Items**	**Day**	**Parity order**			**SE**
		**Nulliparous**	**Primiparous**	**Multiparous**	
Milk yield, kg/day	−0.008 (<0.0001)	6.5b^1^	6.3b	7.2a	0.223
Fat, %	0.0030 (<0.0001)	4.80	4.90	5.02	0.130
Protein, %	0.0010 (<0.0001)	3.21b	3.20b	3.37a	0.055
Lactose, %	0.0001 (0.496)	4.56	4.53	4.56	0.031
Total solids, %	0.004 (<0.0001)	13.73b	13.86b	14.03a	0.163
Fat, kg	0.0003 (<0.0001)	0.316b	0.318b	0.367a	0.010
Protein, kg	−0.0002 (<0.0001)	0.203b	0.203b	0.242a	0.008
Lactose, kg	−0.0004 (<0.0001)	0.286b	0.280b	0.328a	0.011
Total solids, kg	−0.0009 (<0.0001)	0.868b	0.864b	0.932a	0.026
Afternoon/morning^2^	0.0001 (0.786)	0.79	0.71	0.78	0.050
Afternoon/total^2^	0.0001 (0.261)	0.43	0.41	0.42	0.013

1 *Parity order followed by the same letters in the row are statistically equal by dummy variable-based F-test at 5% of probability (significance level)*.

2 *Production ratios afternoon/morning and afternoon/total*.

**Figure 2:**
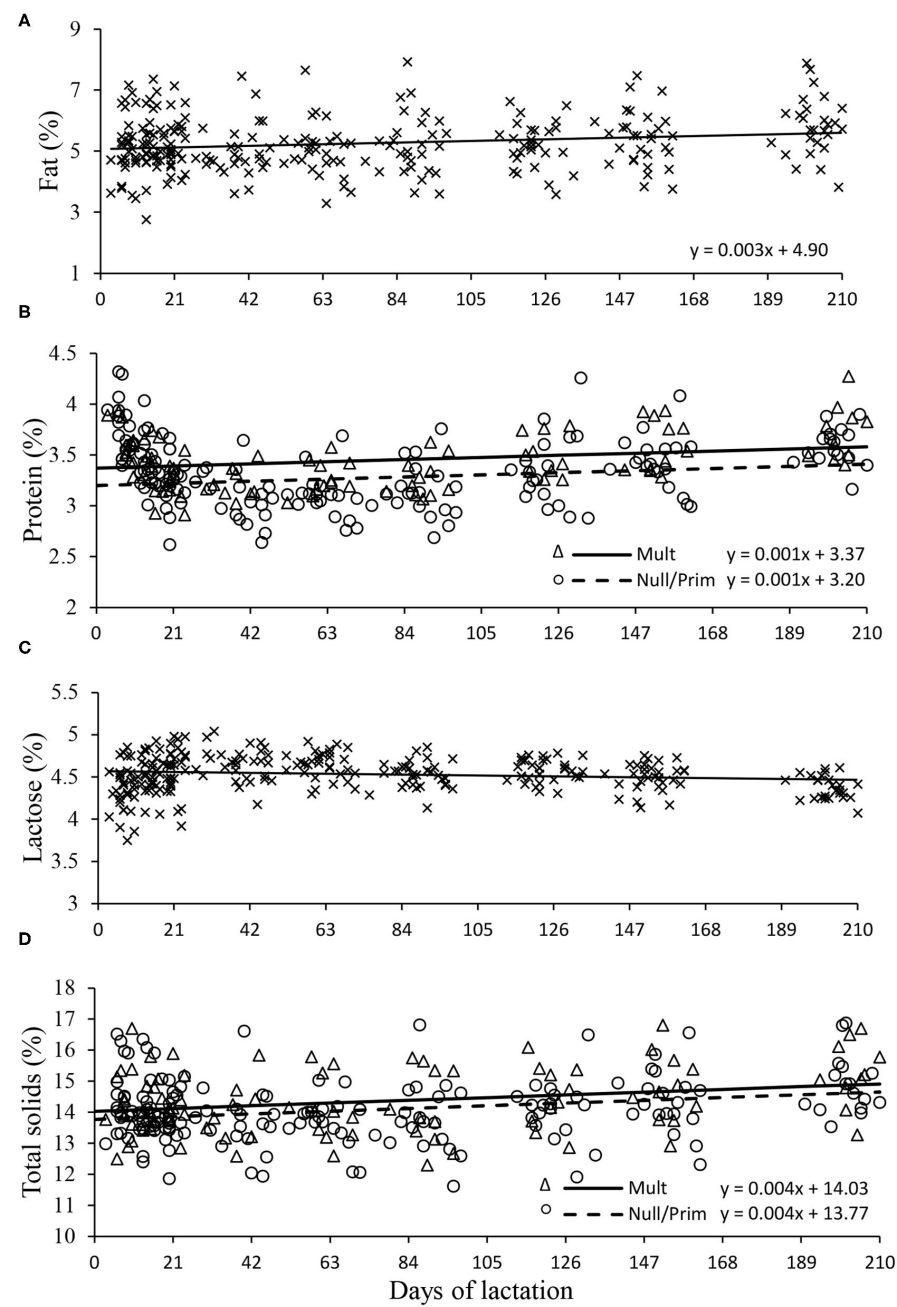
Milk fat **(A)**, protein **(B)**, lactose **(C)**, and total solids **(D)** of Nellore cows according to parity order under grazing. Mult (multiparous), Prim (primiparous), and Null (nulliparous).

Milk fat did not differ between parities (*P* > 0.05); however, there was an effect of days in milking, in which milk fat increased linearly by 0.001% per day (*P* < 0.05; Table 2 and Figure 2A). Milk lactose was on average 4.52% and did not differ between parities or days in milking (*P* > 0.05; Table 2 and Figure 2C).

Milk protein and total solids were different between parities (*P* < 0.05; Table 2), being higher for multiparous cows (0.11 and 0.31%, respectively). Days in milk also affected these variables, as they increased linearly by 0.001 and 0.003% per day, respectively (Figures 2B,D).

However, the yield of milk components expressed in kilogram (fat, protein, lactose, and total solids yield) was all higher for multiparous cows (*P* < 0.05), while nulliparous and primiparous cows displayed similar yields (*P* > 0.05; Table 2).

**Figure 3:**
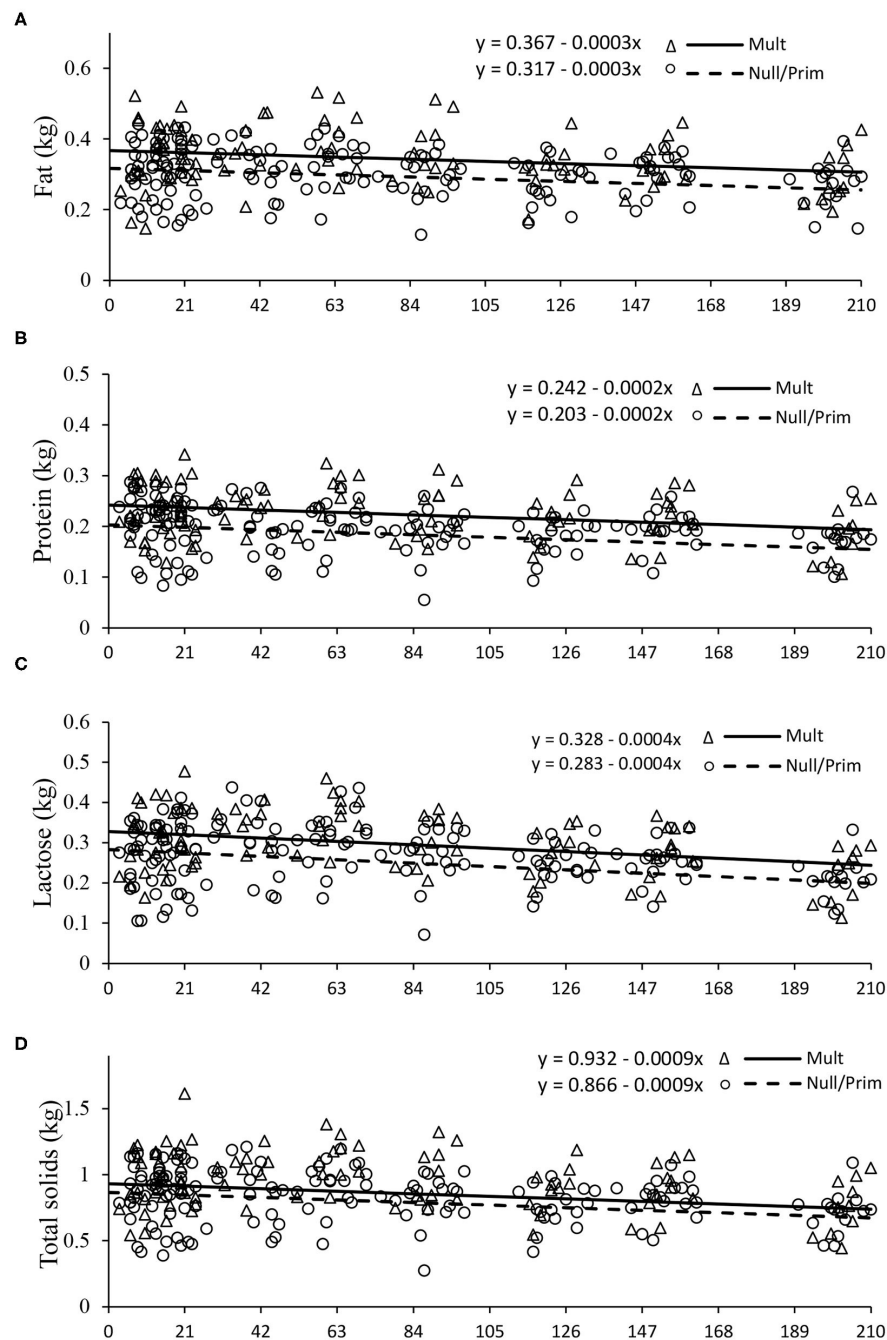
Fat yield **(A)**, protein yield **(B)**, lactose yield **(C)**, and total solids yield **(D)** of Nellore cows according to parity order under grazing. Mult (multiparous), Prim (primiparous), and Null (nulliparous).

The intercepts for fat, protein, lactose, and total solids yield were 0.367, 0.242, 0.328, and 0.932 for multiparous cows and 0.317, 0.283, 0.242, and 0.866 for nulliparous and primiparous cows, respectively, decreasing linearly by 0.0003, 0.0002, 0.0004, and 0.0009 per day, respectively (*P* < 0.05, Figure 3). The afternoon/morning and afternoon/total proportions of milk production were not affected by parity or days of milking (*P* < 0.05), with an average of 0.76 and 0.42, respectively (Table 2).

Based on the milk yield differences between parities, two different groups were used for lactation curve analysis: Mult (multiparous cows) and Null/Prim (both nulliparous and primiparous categories together).

The BR-Corte equation did not estimate the milk yield of the dataset correctly because the intercept and slope did not differ from 0 and 1, respectively (*P* < 0.05). The BR-Corte equation demonstrated overprediction, with 53 and 67% of the MSEP related to model bias Table 3, which shows the need for the development of new equations to estimate the milk yield of grazing Nellore cows.

**Table 3:** Mean (kg) and descriptive statistics of the relationship among the observed and predicted values of milk production of Nellore cows according to parity order.

**Item**	**Multiparous**		**Nulliparous/Primiparous**	
	**OBS^a^**	**BR**	**OBS^a^**	**BR**
Mean	6.60	7.98	5.94	7.98
Standard deviation	1.64	0.60	1.50	0.60
Maximum	10.26	8.67	9.19	8.67
Minimum	2.69	6.82	2.59	6.82
*R* ^b^	-	0.41	-	0.30
CCC^c^	-	0.16	-	0.08
Regression				
Intercept ± SD^d^	-	−2.34 ± 2.04	-	−0.25 ± 1.51
Slope ± SD	-	1.12 ± 0.25	-	0.77 ± 0.18
*P*-value^e^	-	<0.0001	-	<0.0001
MSEP^d^		4.10		6.29
SB	-	1.90	-	4.24
MaF	-	0.00	-	0.01
MoF	-	2.19	-	2.03

a *OBS, observed values; BR, predicted values in BR-Corte (12)*.

b *R, correlation coefficient*.

c *CCC, correlation and concordance coefficient*.

d *SD, standard deviation*.

e *H_0_: b0 = 0 and b1 = 1*.

f *MSEP, mean square error of prediction; SB, square bias; MaF, magnitude of random fluctuation; MoF, model random fluctuation*.

**Figure 4:**
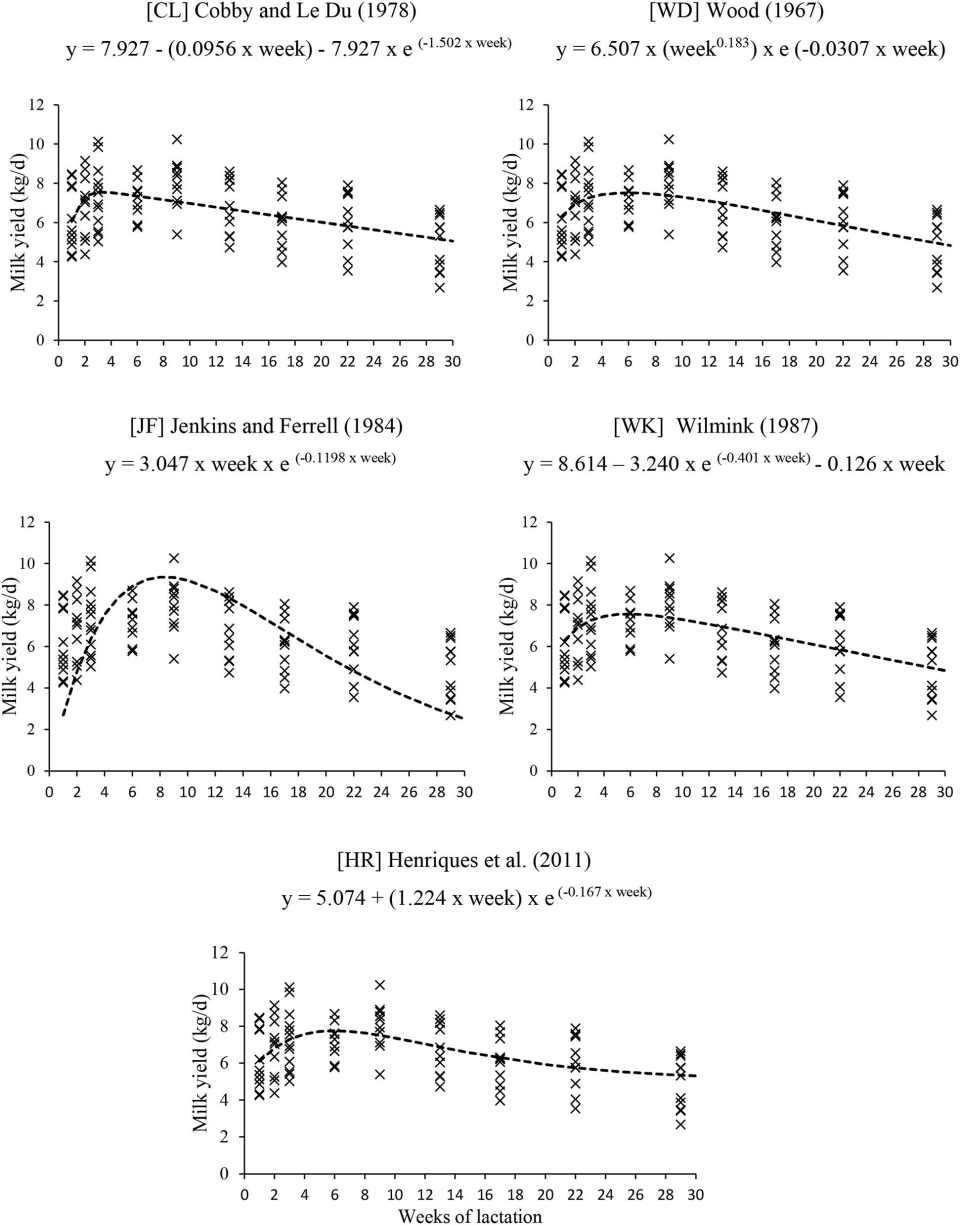
Equations to predict milk yield of multiparous cows under grazing.

**Figure 5:**
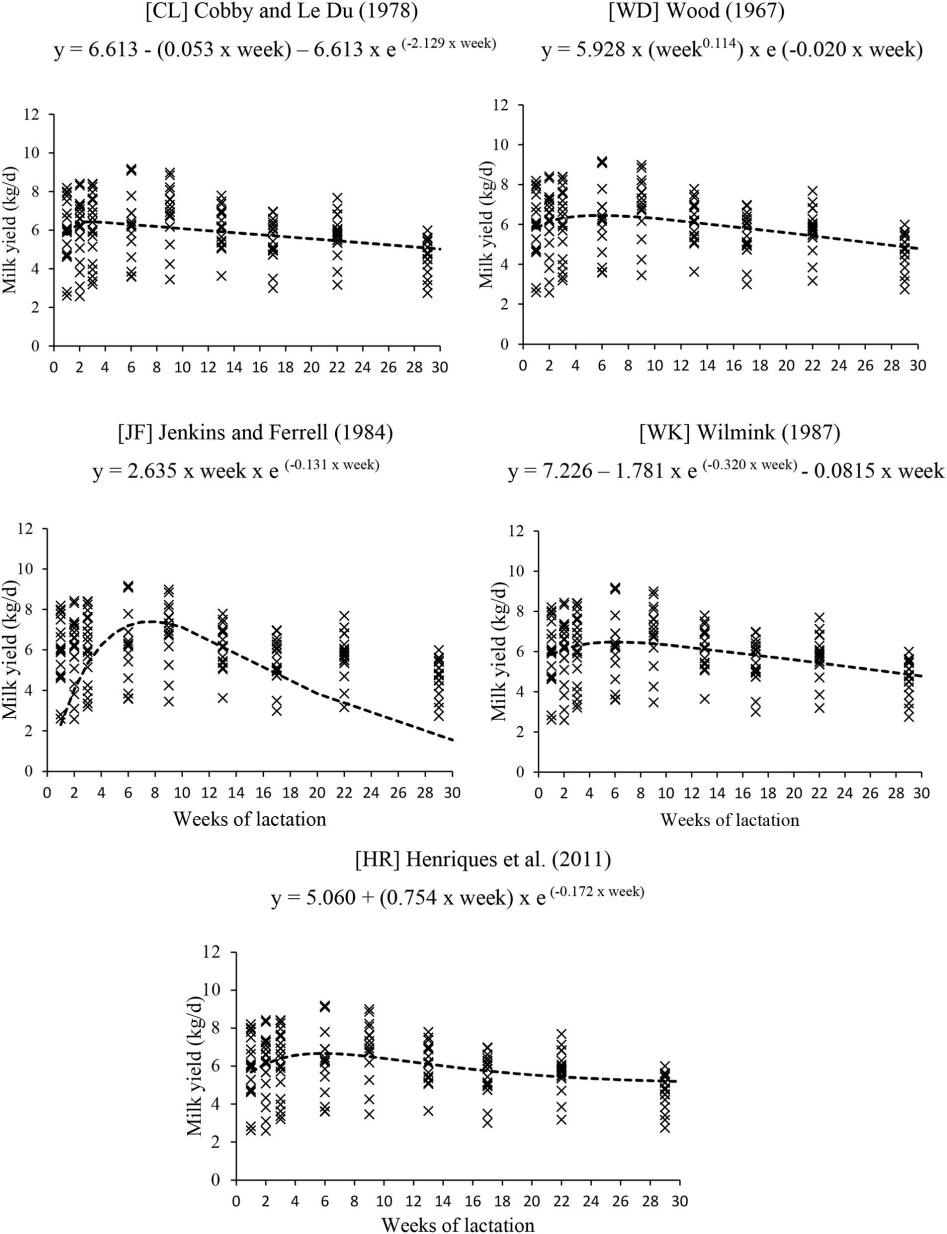
Equations to predict milk yield of nulliparous and primiparous cows under grazing.

Therefore, five non-linear alternative equations forms were fitted to the datasets Mult and Null/Prim: CL, WD, WK, JF, and HR (Figures 4, 5).

The equation JF fitted for Mult and Null/Prim did not estimate the milk yield of the independent data correctly because the intercept and slope did not differ from 0 and 1, respectively. In addition, both equations had the highest AICC and MSEP, showing that the models did not adjust to the independent data (Tables 4, 5).

**Table 4:** Equation parameters and descriptive statistic of the relationship between the observed and predicted values of milk production of multiparous Nellore cows under grazing.

	**OBS^a^**	**WD**	**HR**	**JF**	**WK**	**CL**
***Parameters***						
a	-	6.507	5.074	3.475	8.614	7.927
b	-	0.184	1.224	-	3.240	−0.096
c	-	0.031	0.168	0.120	0.401	1.503
d	-	-	-	-	0.126	-
AICC^b^	-	303.3	305.8	445.6	305.5	309.4
***Descriptive statistics***						
Mean	6.96	6.67	6.75	8.09	6.73	6.70
SD^c^	1.61	0.74	0.77	0.58	0.78	0.77
Maximum	10.3	7.43	7.76	8.67	7.57	7.55
Minimum	3.50	5.00	5.35	6.82	4.97	5.15
Regression						
Intercept ± SD	-	1.90 ± 1.222	2.05 ± 1.200	7.49 ± 0.112	2.08 ± 1.162	2.27 ± 1.177
*P*-value^d^	-	0.122	0.089	<0.0001	0.075	0.065
Slope ± SD	-	0.75 ± 0.182	0.72 ± 0.176	0.09 ± 0.016	0.72 ± 0.171	0.70 ± 0.174
*P*-value^e^	-	0.186	0.122	<0.0001	0.112	0.088
CCC^f^	-	0.256	0.264	0.123	0.272	0.257
MSEP^g^	-	2.36	2.35	10.15	2.35	2.39
SB	-	0.085	0.040	4.038	0.055	0.070
MaF	-	0.031	0.044	5.856	0.046	0.053
MoF	-	2.253	2.270	0.258	2.245	2.273

a *OBS, independent values*.

b *AICC, Akaike's information criterion with correction*.

c *SD, standard deviation*.

d *H_0_: β_0_ = 0*.

e *H_0_: β_1_ = 1*.

f *CCC, correlation and concordance coefficient*.

g *MSEP, mean square error of prediction; SB, square bias; MaF, magnitude of random fluctuation; MoF, model random fluctuation. WD, Wood (24); HR, Henriques et al. (27); JF, Jenkins and Ferrell (26); WK, Wilmink (25); CL, Cobby and Le Du (13)*.

**Table 5:** Equation parameters and descriptive statistics of the relationship between the observed and predicted values of milk production of nulliparous and primiparous Nellore cows under grazing.

	**OBS^a^**	**WD**	**HR**	**JF**	**WK**	**CL**
***Parameters***						
a	-	5.928	5.006	2.630	7.227	6.613
b	-	0.114	0.755	-	1.781	−0.053
c	-	0.020	0.172	0.131	0.320	2.129
d	-		-	-	0.081	-
AICC^b^	-	442.6	446.1	789.2	444.1	452.8
***Descriptive statistics***						
Mean	5.92	6.05	6.83	1.12	6.14	6.31
SD^c^	1.34	0.42	0.72	0.47	0.42	0.50
Maximum	8.80	6.45	7.76	1.72	6.48	6.85
Minimum	2.90	4.87	5.35	0.17	4.86	5.13
Regression						
Intercept ± SD	-	−1.05 ± 1.759	2.24 ± 1.182	5.47 ± 0.378	−0.82 ± 1.75	−0.59 ± 1.509
*P*-value^d^	-	0.549	0.060	<0.0001	0.637	0.694
Slope ± SD	-	1.15 ± 0.290	0.53 ± 0.172	0.08 ± 0.070	1.11 ± 0.280	1.03 ± 0.238
*P*-value^e^	-	0.596	0.008	<0.0001	0.689	0.887
CCC	-	0.203	0.180	0.015	0.199	0.238
MSEP^g^	-	1.58	2.57	24.71	1.59	1.64
SB	-	0.016	0.822	23.066	0.016	0.124
MaF	-	0.004	0.111	0.004	0.002	0.0003
MoF	-	1.560	1.641	1.635	1.571	1.523

a *OBS, independent values*.

b *AICC, Akaike's information criterion with correction*.

c *SD, standard deviation*.

d *H_0_: β_0_ = 0*.

e *H_0_: β_1_ = 1*.

f *CCC, correlation and concordance coefficient*.

g *MSEP, mean square error of prediction; SB, square bias; MaF, magnitude of random fluctuation; MoF, model random fluctuation. WD, Wood (24); HR, Henriques et al. (27); JF, Jenkins and Ferrell (26); WK, Wilmink (25); CL, Cobby and Le Du (13)*.

The HR equation did not estimate the milk yield of the independent data for the Null/Prim equation because the slope did not differ from 1 (*P* < 0.05; Table 5). Even though the HR equation fitted well to the Mult data, we did not consider it as a plausible equation form for use, as it did not adjust also to the Null/Prim dataset.

The equations of WD, WK, and CL had the best estimate of milk yield and description of the lactation curve of grazing Nellore cows for both Mult and Null/Prim datasets (Tables 4, 5). The equations had very similar AICC and MSEP, with 96% for Mult and around 92 to 96% for Null/Prim of the MSEP associated with random error (MoF). All three equations were similar in estimate mean milk yield; hence, the choice of any of those would not impact negatively the estimate of milk yield.

The estimated milk yield at the peak of production and time until peak for Mult and Null/Prim according to equations form are as follows:

WD = 7.49 kg at 5.90 weeks for Mult and 6.45 kg at 5.72 weeks for Null/Prim.
WK = 7.56 kg at 5.82 weeks for Mult and 6.08 kg at 6.47 weeks for Null/Prim.
CL = 7.55 kg at 3.30 weeks for Mult and 6.45 kg at 2.62 weeks for Null/Prim.

**Figure 6:**
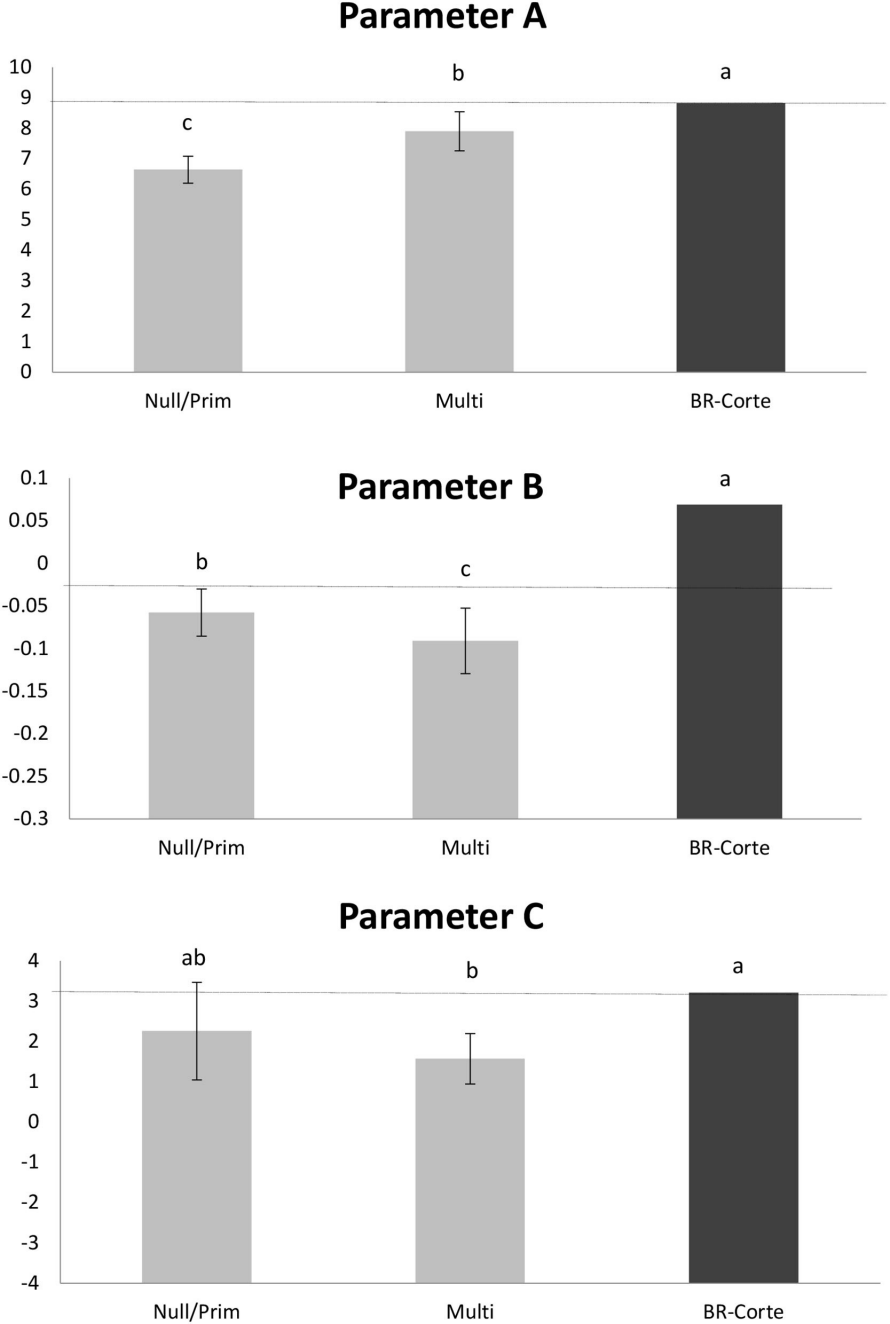
Identity model analysis using a confidence interval approach to compare the parameters (*Y*_*t*_ = *a* + *bt* – *e*^−*ct*^) of the three equations based on the CL model: Multi, Null/Prim, and BR-Corte. Different letters represent significant differences between parameter values.

The parameters *a, b*, and *c* of the CL equation generated for Mult and Null/Prim differ from the BR-Corte parameters (Figure 6).

## Discussion

Nulliparous and primiparous cows are in a different physiological state than multiparous cows since they are not physically mature at this age (28). Therefore, a difference in milk production was expected; however, the magnitude of the differences was not known.

Our outcome reveals an average milk yield that was approximately 11% lower (−0.70 kg/day) for nulliparous and primiparous cows than for multiparous Nellore cows. These results are consistent with other studies evaluating *Bos taurus* dairy (15, 16) and beef cows (6, 29). However, the range of the differences in milk yield according to age or parity, as well as the lactation peak and persistence, may vary between species (such as *B. taurus* and *Bos indicus*) and breed crosses.

In the *Nutrient Requirements of Beef Cattle*, NASEM (2016), the equation used to predict milk yield of beef cows includes an age coefficient that represents 26 and 12% less milk yield for cows of ≤ 2 years and >2 but ≤ 3 years, respectively (6). Also, according to NASEM, the peak lactation of beef cows (based on a wide variety of *B. taurus* breed and breed crosses) occurs approximately at 8.5 weeks (6). In lactating dairy cows, milk production usually peaks at 4 to 8 weeks postpartum (16). In contrast to both studies, for Nellore cows, we found that the peak of lactation is substantially earlier (around 3 to 6 weeks) than that observed for *B. taurus* dairy and beef cows. There is no mention of differences in the milk yield and time to peak of lactation according to the parity or age of Nellore cows in the BR-Corte editions (12, 14).

Greater milk production of multiparous cows may be explained by the largest balance of net energy for milk production, since this category of nutritional requirements is only for maintenance and milk production, unlike young cows that still have nutritional growth requirements. Also, as nulliparous cows still require nutrients for their continued growth, the mammary gland is not completely developed, and the capacity of milk production is reduced compared to multiparous cows. In dairy cows, although the underlying mechanisms are not well understood, several indicators suggest that the mammary gland is more metabolically active in multiparous cows (i.e., higher expression of genes related to metabolic activity) than in primiparous cows, especially at the onset and peak of lactation (30). This observation suggests, at least in part, that the lower milk production observed in primiparous cows could be related to a lower density of secretory cells. Generally, multiparous animals have a higher milk yield but lower lactation persistency than younger animals (30–32). In this experiment, besides the lower milk yield, the lactation curve of nulliparous and primiparous cows was indeed much flatter than that of multiparous cows.

The average milk composition of Nellore cows in BR-Corte (12) was 15.0% total solids, 4.58% lactose, and 5.61% fat during all lactation periods. However, milk protein increased during the lactation period from 3.57% (4th week) to 3.97% (28th week). In the present study, all milk components changed during the lactation period except for lactose. Total solids, protein, and fat increased linearly throughout lactation. However, when milk composition is expressed in kilograms per day, all components' yield decreased throughout lactation because the percentage of milk yield reduction was greater than the solids increase. The greatest fat percentage value observed in BR-Corte (12) may be related to a higher-energy diet since the cows were maintained in a feedlot system and fed corn silage, which also impacted the milk total solids values. Milk composition can vary according to breed and diet; however, in general, data from previous studies show a percentage of milk fat ranging from 4.5 to 5% for Nellore cows (10, 11, 33).

Reduced milk protein and total solids for nulliparous cows were expected. They may be explained by the differences in protein metabolic status since nulliparous cows have lower blood albumin and total protein than multiparous beef (17) and dairy cows (34, 35). These differences are even higher when milk components were expressed in kilograms since parities also differ in milk yield.

Over the years, several models and approaches have been used to describe the lactation curve of cattle (13, 24–27, 36, 37). The equation most widely used for describing the lactation curve of dairy cows is the model proposed by WD (24); however, its use for beef cattle milk production has been limited since it demands a relatively large number of data points to fit the equation. Therefore, NASEM used a similar equation proposed by JF (26) that requires fewer data points (6).

In the study of HR (27), the best equations to estimate the milk yield of Nellore cows were based on WD (24) and a modification of the JF (26) equation. However, the method of milk collection was weigh–suckle–weigh, then multiplying the morning yield by 2 to estimate daily milk yield, which has been criticized due to data variation and wrong daily estimates. The model that fits better for describing a lactation curve for Nellore cows, according to the BR-Corte (12), is the one proposed by CL (13) in which, after peak, milk yield tends to decline linearly. Although the equation presented in BR-Corte (12) provides a better estimation than the past editions, there are still limitations on its database.

Studies regarding milk production of Nellore cows are often conducted in pens and using diets that mischaracterize the range cattle system, as well as methods of milking that can overestimate or underestimate the milk production depending on the approach. Therefore, the BR-Corte model overprediction was expected because the equation was based on a study developed in a feedlot system using corn silage as a roughage source (38). Although the experiment validated the equation with independent data of studies with grazing Nellore cows, in all of these studies (including the observed values), the cows were milked once in the morning, and then this value was doubled to estimate daily production or extrapolated the morning milk yield for a 24-h period. We believe that this overestimation is also related to the equivocated estimation of milk yield based on only one milking because the morning and afternoon yields contribute to the total daily production in different ratios (10, 39). Based on this rationale, the development of new equations that correctly estimate milk production on a grazing range system was necessary. Those explanations are supported by the analysis of model identity that showed that the parameters of the CL equations created compared with the BR-Corte were indeed different, even for the multiparous equation, which used the same animal category as the BR-Corte database.

The equations of WD, WK, and CL were the best in estimating milk yield and describing the lactation curve of both Mult and Null/Prim datasets. All three equations were similar in estimates of mean milk yield and milk production upon lactation peak. The only difference between the equations is the occurrence peak of production, in which the CL equation estimates an earlier peak of production than the WD and WK equation forms. In terms of using milk yield information for energy and protein requirements, choosing any of those would not negatively impact the data estimates.

In this experiment, morning and afternoon yields indeed contributed at different ratios to the total daily production. However, the values are not consistent with those of other studies (10, 39). In addition, we did not find any effect of days in milking for these ratios. Thus, it can be assumed that only one ratio for the whole lactation is needed to estimate total daily milk production, based only on the morning milking.

## Conclusions

Parity influences the milk production and composition of beef cows. Milk yield can be estimated based on WD, WK, and CL equation forms, using different equations for multiparous (4–6 years) and nulliparous/primiparous (2–3 years) cows, with an estimated average peak of milk production of 7.5 and 6.3 kg, respectively, and time until peak ranging from 3 to 6 weeks.

## Data Availability Statement

The raw data supporting the conclusions of this article will be made available by the authors, without undue reservation.

## Ethics Statement

The animal study was reviewed and approved by the Animal Care and Use Committee at the Universidade Federal de Viçosa, Brazil (protocol CEUAP-UFV 120/2018) approved all animal care and handling procedures.

## Author Contributions

MF conceived the study, carried out the experimental trial, performed the statistical analysis and chemical analyses, and wrote the manuscript. LR contributed to draft the manuscript and coordinate the research group. IR carried out the experimental trial and contributed to the draft of the manuscript. SF contributed to data interpretation and to draft the manuscript. FS, ED, and MP contributed to designing the experiment, statistical analysis, and drafting the manuscript. All authors read and approved the final manuscript.

## Funding

This research was supported by funding from CNPq (Conselho Nacional de Desenvolvimento Científico e Tecnológico), CAPES (Coordenação de Aperfeiçoamento de Pessoal de Nível Superior), FAPEMIG (Fundação de Amparo à Pesquisa de MG), and INCT-CA (Instituto Nacional de Ciência e Tecnologia de Ciência Animal). The funding body had no role in the design of the study and collection, analysis, and interpretation of data and in writing the manuscript.

## Conflict of Interest

The authors declare that the research was conducted in the absence of any commercial or financial relationships that could be construed as a potential conflict of interest.

## Publisher's Note

All claims expressed in this article are solely those of the authors and do not necessarily represent those of their affiliated organizations, or those of the publisher, the editors and the reviewers. Any product that may be evaluated in this article, or claim that may be made by its manufacturer, is not guaranteed or endorsed by the publisher.
